# Antiproliferative factor decreases Akt phosphorylation and alters gene expression via CKAP4 in T24 bladder carcinoma cells

**DOI:** 10.1186/1756-9966-29-160

**Published:** 2010-12-10

**Authors:** Hanief M Shahjee, Kristopher R Koch, Li Guo, Chen-Ou Zhang, Susan K Keay

**Affiliations:** 1 Division of Infectious Diseases, Department of Medicine, University of Maryland School of Medicine, Baltimore, Maryland, USA; 2 Staff physician, Medical Service, Department of Veterans Affairs Medical Center, Baltimore, Maryland, USA

## Abstract

**Background:**

Urinary bladder cancer is a common malignancy worldwide, and outcomes for patients with advanced bladder cancer remain poor. Antiproliferative factor (APF) is a potent glycopeptide inhibitor of epithelial cell proliferation that was discovered in the urine of patients with interstitial cystitis, a disorder with bladder epithelial thinning and ulceration. APF mediates its antiproliferative activity in primary normal bladder epithelial cells via cytoskeletal associated protein 4 (CKAP4). Because synthetic asialo-APF (*as*-APF) has also been shown to inhibit T24 bladder cancer cell proliferation at nanomolar concentrations *in vitro*, and because the peptide segment of APF is 100% homologous to part of frizzled 8, we determined whether CKAP4 mediates *as*-APF inhibition of proliferation and/or downstream Wnt/frizzled signaling events in T24 cells.

**Methods:**

T24 cells were transfected with double-stranded siRNAs against CKAP4 and treated with synthetic *as*-APF or inactive control peptide; cells that did not undergo electroporation and cells transfected with non-target (scrambled) double-stranded siRNA served as negative controls. Cell proliferation was determined by ^3^H-thymidine incorporation. Expression of Akt, glycogen synthase kinase 3β (GSK3β), β-catenin, p53, and matrix metalloproteinase 2 (MMP2) mRNA was determined by quantitative reverse transcriptase polymerase chain reaction (qRT-PCR). Akt, GSK-3β, MMP2, β-catenin, and p53 protein expression, plus Akt, GSK-3β, and β-catenin phosphorylation, were determined by Western blot.

**Results:**

T24 cell proliferation, MMP2 expression, Akt ser473 and thr308 phosphorylation, GSK3β tyr216 phosphorylation, and β-catenin ser45/thr41 phosphorylation were all decreased by APF, whereas p53 expression, and β-catenin ser33,37/thr41 phosphorylation, were increased by APF treatment in non-electroporated and non-target siRNA-transfected cells. Neither mRNA nor total protein expression of Akt, GSK3β, or β-catenin changed in response to APF in these cells. In addition, the changes in cell proliferation, MMP2/p53 mRNA and protein expression, and Akt/GSK3β/β-catenin phosphorylation in response to APF treatment were all specifically abrogated following CKAP4 siRNA knockdown.

**Conclusions:**

Synthetic *as*-APF inhibits cell proliferation in T24 bladder carcinoma cells via the CKAP4 receptor. The mechanism for this inhibition involves regulating phosphorylation of specific cell signaling molecules (Akt, GSK3β, and β-catenin) plus mRNA and protein expression of p53 and MMP2.

## Background

Bladder cancer is the second most common genitourinary malignancy and the fourth most common malignancy in men in the United States, causing over 12,000 deaths annually [1]. Although seventy percent of cases are diagnosed in the superficial stage, up to 30% can present with or develop muscle-invasive disease, and long term outcomes for patients with advanced bladder cancer remain poor [2,3]. Additional treatments that prevent or control the progression of bladder carcinoma are therefore sorely needed.

Altered expression of certain genes commonly found in human carcinomas are also found in bladder cancer, including decreased expression of E-cadherin [4-8] and the tumor suppressors p53 and p21 [9-11], with increased expression of heparin-binding epidermal growth factor-like growth factor (HB-EGF) [12]. Of these abnormalities, decreased E-cadherin and increased HB-EGF expression appear to be particularly closely associated with increased tumor progression, cell proliferation, and/or metastasis [5-8,12-15]. Therapies aimed at controlling the aberrant expression of genes associated with tumor progression and metastasis in bladder carcinoma cells may be helpful for controlling disease.

Our laboratory previously discovered a natural antiproliferative factor (APF) [16-18] that profoundly inhibits bladder epithelial cell proliferation [19,20], upregulates E-cadherin [21], p53 and p21 [22] expression, and inhibits the production of other cell proteins including HB-EGF [17,20,21,23]. APF is secreted specifically by bladder epithelial cells from patients with interstitial cystitis (IC), a chronic bladder disorder characterized by bladder epithelial thinning and/or ulceration [24-26]. APF is a low molecular weight frizzled 8-related glycopeptide that inhibits both normal and IC bladder epithelial cell proliferation via cytoskeleton associated protein 4 (CKAP4, also known as CLIMP-63 and ERGIC-63) [27], a type II transmembrane receptor [28] whose palmitoylation appears to be required for mediating APF activity in HeLa cells [29]. Synthetic asialo-APF (*as*-APF) inhibits T24 bladder carcinoma cell proliferation *in vitro *at low (nanomolar) concentrations similar to those required for inhibition of normal bladder epithelial cell proliferation [19]. However, neither the role of CKAP4 in regulation of bladder carcinoma cell proliferation, nor its role in mediating APF activity in bladder carcinoma cells, has yet been studied.

Therefore, to better understand the mechanism by which APF regulates T24 bladder carcinoma cell proliferation, we determined the effect of *as*-APF on the expression or activation of enzymes involved in wingless-int (Wnt)/frizzled signaling (including AKR-transforming enzyme (Akt), glycogen synthase kinase-3 beta (GSK3β), β-catenin, and matrix metalloprotease 2 (MMP2), as well as the role of CKAP4 in mediating *as*-APF activity in T24 cells.

## Methods

### Cell Culture

T24 human urinary bladder cancer cells (ATCC HTB-4) were grown to 60-80% confluence in McCoy's 5A medium (Invitrogen, Carlsbad, CA) containing 10% heat-inactivated fetal bovine serum (FBS), 1% antibiotic/antimycotic solution, 1% L-glutamine (all from Sigma, St. Louis, MO) and 2.2 grams/L sodium bicarbonate (Invitrogen), in a 37°C/5% CO_2 _atmosphere.

### siRNA Transfection

Double-stranded siRNA corresponding to nucleotides 594-616 of CKAP4 (5'-AACUUUUGAGUCCAUCUUGAGAA-3' sense strand) and a scrambled double-stranded negative control siRNA (5'-AAUUCUGUAUGCUACCUGUAGAA-3' sense strand) were prepared by preincubating single-stranded sense and antisense strands prepared with double A overhangs in serum-free McCoy's 5A medium at 37°C for 1 hour. T24 human bladder cancer cells were trypsinized for 10 minutes at room temperature, centrifuged in growth medium (as defined above), and the cell pellet was resuspended in serum-free medium at a density of 1 × 10^6 ^cells/ml. Two hundred microliters of the cell suspension were then transferred to a sterile 2-mm cuvette with 14 μg of CKAP4 siRNA, scrambled non-target siRNA, or no siRNA, and electroporated at 160 V/500 microfarad capacitance using a Bio-Rad Gene Pulser Xcell. The cells were then immediately transferred to T75 cell culture flasks (Corning Incorporated, Corning, NY) (for extraction of RNA and protein) or to 96 well tissue culture plates (Corning Incorporated) (for the cell proliferation assay) and incubated in growth medium overnight in a 37°C/5% CO_2 _atmosphere.

### APF Treatment (for RNA and Protein Extraction)

Following overnight incubation in growth medium, transfected T24 human bladder cancer cells were further incubated with serum-free McCoy's 5A medium for the next 24 hours, after which they were treated with 500 nM *as*-APF or 500 nM inactive nonglycosylated peptide control (both from PolyPeptide Laboratories, Incorporated, San Diego, CA). Cells were then incubated for an additional 48 hrs in a 37°C/5% CO_2 _atmosphere prior to RNA and protein extraction.

### RNA Extraction

Following cell incubation with *as*-APF or its control peptide/diluent, the culture medium was removed, T24 cells were washed with 1× PBS, and RNA was extracted using the RNeasy Plus Mini Kit (Qiagen, Valencia, CA) according to the manufacturer's instructions. RNA concentration was measured at 260 nM in a UV/VIS spectrophotometer from Perkin Elmer. Extracted RNA was stored at -80°C.

### Protein Extraction

Cell culture medium was removed from duplicate flasks, T24 cells were scraped into ice-cold PBS, and the cell slurry was centrifuged at 4°C for 5 minutes at 2000 rpm. Supernatant was then removed and the pellet was washed with ice-cold PBS and centrifuged again at 4°C for 5 minutes at 2000 rpm. This pellet was then resuspended in ice-cold RIPA buffer (Upstate Cell Signaling Solutions, Temecula, CA) containing Complete Protease Inhibitor Cocktail (Roche, Indianapolis, IN) and centrifuged at 14,000 rpm for 15 minutes at 4°C. Supernatant containing total cell protein was collected and stored at -80°C.

### ^3^H-Thymidine Cell Proliferation Assay

Cell proliferation was measured by ^3^H-thymidine incorporation into T24 human bladder cancer cells, plating 1.5 ×10^3 ^cells/well onto a 96-well cell culture plate (Corning Incorporated), in 150 μL/well McCoy's 5A medium containing 10% heat inactivated FBS, 1% antibiotic/antimycotic solution, 1% L-glutamine, and plus 2.2 grams/L sodium bicarbonate. The next day, cell growth medium was removed and replaced with 100 μl serum-free McCoy's medium. On the third day, synthetic *as*-APF was resuspended in acetonitrile/distilled water (1:1) and applied to the cells in serum-free McCoy's medium at varying concentrations; cell controls received acetonitrile/distilled water diluted in serum-free McCoy's medium (same final concentration of diluent). Cells were then incubated at 37°C in a 5% CO_2 _atmosphere for an additional 48 hours, after which they were labeled with 1 μCi per well ^3^H-thymidine at 37°C in a 5% CO_2 _atmosphere for 4 hours. The cells were then treated with trypsin-EDTA (Invitrogen), insoluble cell contents harvested and methanol-fixed onto glass fiber filter paper, and the amount of radioactivity incorporated determined using a Beckman scintillation counter. Significant inhibition of ^3^H-thymidine incorporation was defined as a decrease in cpm of >2 SD from the mean of control cells for each plate.

### Real-time qRT-PCR

Gene expression was determined using SYBR^® ^Green based real-time RT-PCR, QuantiTect^® ^primers and reagents (Qiagen) and a Roche 480 LightCycler. Samples were tested in triplicate runs, and specific mRNA levels quantified and compared to mRNA levels for β-actin or GAPDH using Roche LC480 real-time PCR analysis software (version 1.5.0). Predetermined optimal concentrations of RNA were used for each set of primers. p53 (QT00060235), Akt (QT00085379), GSK3β (QT00057134), β-catenin (QT00077882), MMP2 (QT00088396), GAPDH (QT01192646), and β-actin (QT1680476) primer sets were obtained from Qiagen. p53 served as a standard control for APF activity, while GAPDH and β-actin served as standard controls for the qRT-PCR procedure.

### SDS Polyacrylamide Gel Electrophoresis and Western Blot Assay

Specific protein expression or phosphorylation was determined by Western blot. Protein concentration was measured using a Folin reagent-based protein assay kit (Bio-Rad, Hercules, CA). Solubilized cell proteins were incubated for 10 min at 70°C in sample reducing buffer, each lane was loaded with 30 μg of protein, and proteins were separated by electrophoresis using 4-12% NuPAGE NOVEX BisTris polyacrylamide gels (Invitrogen) in MOPS/SDS running buffer (Invitrogen), according to the manufacturer's instructions. Proteins were then transferred to nitrocellulose membranes (Invitrogen) according to the NuPAGE gel manufacturer's protocol for Western transfer (30 V constant voltage for 1 h). Following protein transfer, the nitrocellulose membranes were blocked with 5% nonfat dry milk in TBS-T buffer (Tris-buffered saline, pH 7.4, with 0.1% Tween 20) and incubated overnight at 4°C in TBS-T buffer containing mouse monoclonal anti-CKAP4 ("anti-CLIMP-63," clone G1/296) (Alexis Biochemical, Plymouth Meeting, PA), anti-p53 (Calbiochem, San Diego, CA), anti-GSK3β (BD Biosciences, San Jose, CA), anti-phosphoGSK3β (tyr 216) (BD Biosciences), or anti-β actin (Sigma) antibodies; or rabbit polyclonal anti-MMP2, anti-Akt, anti-phosphoAkt (ser473/thr308), anti-phosphoGSK3β (ser9), anti-β-catenin, anti-phosphoβ-catenin (ser 33,37/thr 41), or anti-phosphoβ-catenin (ser 45/thr 41) (all obtained from Cell Signaling Technology, Danvers, MA). When more than one antibody was used for binding to proteins on a single membrane, the membrane was stripped between antibody incubations using Restore PLUS Western blot stripping buffer (Pierce, Rockford, IL) according to the manufacturer's instructions. The membranes were subsequently washed three times with TBS-T, incubated with horseradish peroxidase-conjugated goat anti-mouse or goat anti-rabbit IgG secondary antibodies (Santa Cruz Biotechnology, Santa Cruz, CA) for 1 h at room temperature, and developed with ECL chemiluminescence Reagent (Amersham Biosciences, Piscataway, NJ). p53 expression served as a positive control for APF activity; β-actin expression served as a standard control for the Western blot procedure.

### Statistical Analysis

Significant inhibition of ^3^H-thymidine incorporation was defined as a mean decrease in cpm of ± 2 SD from the mean of control cells for each plate. Crossover point analysis was performed for qRT-PCR data, and mRNA copy number for each gene was quantified relative to β-actin; this value is expressed as mean ± standard error of the mean (SEM) for duplicate runs performed on three separate occasions. The significance of the difference between mean values was determined by an analysis of variance with p < .05 considered significant.

## Results

### siRNA knockdown of CKAP4 expression inhibits APF antiproliferative activity in T24 bladder carcinoma cells

**Figure 1:**
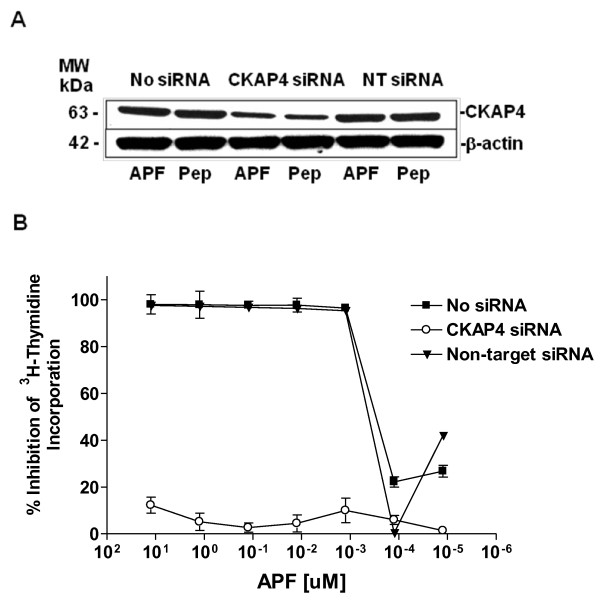
**CKAP4 knockdown in T24 cells**. **A**, Western blot analysis of CKAP4 protein expression in cells electroporated in the presence of no siRNA (Lanes 1 and 2), CKAP4 siRNA (Lanes 3 and 4), or scrambled non-target (NT) siRNA (Lanes 5 and 6), and treated with *as*-APF (APF) or its inactive control peptide (Pep). β-actin served as a standard control. **B**, Inhibition of ^3^H-thymidine incorporation by *as*-APF (APF) in cells electroporated with no siRNA, CKAP4 siRNA, or non-target siRNA. Results are shown as percent inhibition of ^3^H-thymidine incorporation compared to control cells that did not receive *as*-APF treatment. Experiment was performed in triplicate twice.

To determine whether APF activity was mediated by CKAP4 in T24 cells, expression of this receptor was knocked down by double-stranded siRNA transfection via electroporation. Non-target (scrambled) siRNA was used to confirm the specificity of CKAP4 knockdown, and untreated cells served as negative controls for the electroporation procedure. Decreased CKAP4 protein expression following CKAP4 siRNA transfection was confirmed by Western blot (Figure 1A). As shown in Figure 1B, dose-dependent inhibition of T24 cell proliferation by submicromolar concentrations of *as*-APF was specifically and significantly decreased following CKAP4 knockdown (p < .001 for comparison of CKAP siRNA-treated cells compared to both controls at concentrations ≥ 1.25 nM), indicating the importance of this receptor for mediating APF antiproliferative activity in T24 bladder carcinoma cells.

### APF increases p53 tumor suppressor gene expression via CKAP4 in T24 cells

**Figure 2:**
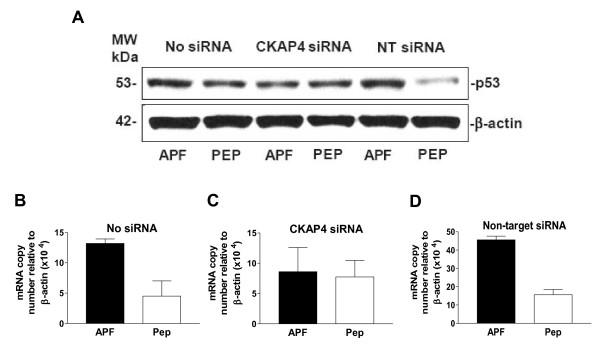
**p53 expression in T24 bladder cancer cells**. **A**, Western blot analysis of p53 protein expression in cells electroporated in the presence of no siRNA (Lanes 1 and 2), CKAP4 siRNA (Lanes 3 and 4), or scrambled non-target (NT) siRNA (Lanes 5 and 6), and treated with *as*-APF (APF) or its inactive control peptide (Pep). *β*-actin served as a standard control. **B**, Quantitative real time RT-PCR analysis of p53 mRNA expression in T24 cells electroporated with no siRNA, **C**, CKAP4 siRNA, or **D**, non-target siRNA, and then treated with *as*-APF (APF) or its inactive control peptide (Pep). Each experiment was performed in duplicate on at least three separate occasions. Data are expressed as mean ± SEM.

HPLC-purified native APF was previously shown to significantly decrease cell cycle transit and increase p53 expression in both normal human urothelial cells and T24 bladder carcinoma cells *in vitro*, while p53 knockdown decreased the antiproliferative effects of APF [22]. To determine whether CKAP4 mediated APF's stimulation of p53 expression, T24 cells were treated with 500 nM synthetic *as-*APF or its inactive peptide control and the effects on p53 mRNA and protein expression examined. As shown in Figure 2A, p53 protein expression was increased in APF-treated (as compared to control peptide-treated) nontransfected cells. Similarly, p53 protein expression was also increased in response to APF in cells transfected with non-target siRNA, whereas p53 levels changed less in response to APF following CKAP4 knockdown (Figure 2A). qRT-PCR also showed significantly increased p53 mRNA expression following APF treatment of nontransfected or non-target siRNA-transfected, but not CKAP4 siRNA-transfected, cells (Figure 2B-D) (p < .01 for both nontransfected and non-target transfected cells, and target gene mRNA relative to β-actin or GAPDH mRNA; data shown for normalization to β-actin expression, only). These findings indicate that CKAP4 also mediates the effects of APF on p53 mRNA and protein expression in T24 cells.

### Decreased Akt (serine 473 and threonine 308) phosphorylation following APF treatment of T24 cells

**Figure 3:**
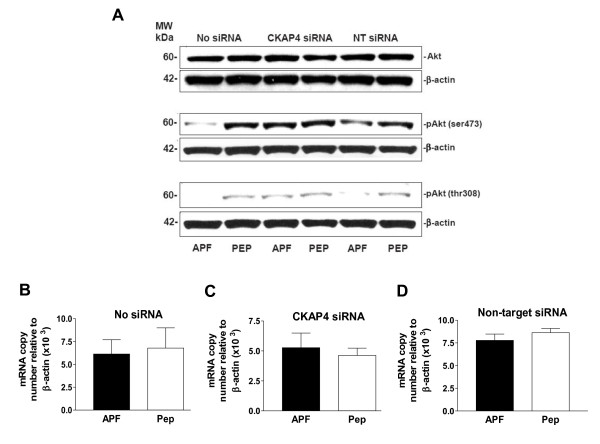
**Akt phosphorylation activity in T24 bladder cancer cells**. **A**, Western blot analysis of Akt protein expression and phosphorylation in cells electroporated in the presence of no siRNA (Lanes 1 and 2), CKAP4 siRNA (Lanes 3 and 4), or scrambled non-target (NT) siRNA (Lanes 5 and 6), and treated with *as*-APF (APF) or its inactive control peptide (Pep). *β*-actin served as a standard control. **B**, Quantitative real time RT-PCR analysis of Akt mRNA expression in T24 cells electroporated with no siRNA, **C**, CKAP4 siRNA, or **D**, non-target siRNA, and then treated with *as*-APF (APF) or its inactive control peptide (Pep). Each experiment was performed in duplicate on at least three separate occasions. Data are expressed as mean ± SEM.

To understand whether Wnt/frizzled signaling might play a role in mediating APF activity in T24 cells, we determined the effect(s) of *as*-APF treatment on Akt expression and serine/threonine phosphorylation in nontransfected, non-target siRNA-transfected, and CKAP4 siRNA-transfected cells. As shown in Figure 3A, APF treatment caused decreased Akt serine 473 (ser473) and threonine 308 (thr308) phosphorylation in nontransfected and non-target siRNA transfected cells, whereas there was no apparent change in phosphorylation at either site in CKAP4 siRNA-transfected cells. However, APF treatment did not appear to affect total Akt protein (Figure 3A) or Akt mRNA (Figure 3B-D) expression, regardless of transfection status (p > .05 for all PCR comparisons, including target gene mRNA relative to β-actin or GAPDH mRNA; data shown for normalization to β-actin expression, only). These findings indicate a potential role for inhibition of Akt activation in CKAP4-mediated APF antiproliferative activity.

### Decreased GSK3β (tyrosine 216) and β-catenin (serine 45/threonine 41) phosphorylation, but increased β-catenin (serine 33, 37/threonine 41) phosphorylation, in response to APF

**Figure 4:**
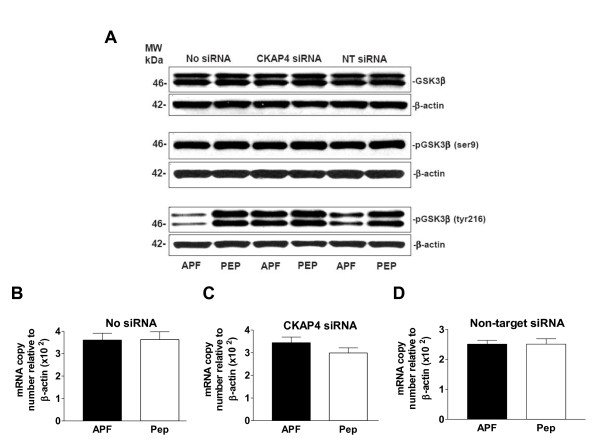
**GSK3β tyr216 phosphorylation activity in bladder cancer cells**. **A**, Western blot analysis of GSK3β protein expression and phosphorylation in cells electroporated in the presence of no siRNA (Lanes 1 and 2), CKAP4 siRNA (Lanes 3 and 4), or scrambled non-target (NT) siRNA (Lanes 5 and 6), and treated with *as*-APF (APF) or its inactive control peptide (Pep). β-actin served as a standard control. **B**, Quantitative real time RT-PCR analysis of GSK3β mRNA expression in T24 cells electroporated with no siRNA, **C**, CKAP4 siRNA, or **D**, non-target siRNA, and then treated with *as*-APF (APF) or its inactive control peptide (Pep). Each experiment was performed in duplicate on at least three separate occasions. Data are expressed as mean ± SEM.

In Wnt signaling pathways, Akt phosphorylation/activation stimulates GSK3β serine 9 (ser9) phosphorylation, leading to its inactivation, which in turn inhibits β-catenin ubiquitination and degradation [30]. We therefore determined whether APF-induced decreased Akt phosphorylation lead to changes in GSK3β and β-catenin phosphorylation in T24 bladder carcinoma cells. Although GSK3β ser9 phosphorylation may have been minimally decreased in APF-treated nontransfected and non-target siRNA-transfected cells response to APF, GSK3β tyrosine 216 (tyr216) phosphorylation was clearly decreased following APF treatment of these same cells (but unchanged in CKAP4 siRNA-transfected cells) (Figure 4A). Again, APF treatment did not appear to affect total GSK3β protein (Figure 4A) or mRNA (Figure 4B-D) expression, regardless of transfection status (p > .05 for all PCR comparisons, including target gene mRNA relative to β-actin or GAPDH mRNA; data shown for normalization to β-actin expression, only). These findings indicate that APF induces changes in GSK3β phosphorylation via CKAP4, but further suggest that APF does not mediate its antiproliferative activity in T24 cells merely by inhibiting canonical Wnt/frizzled signaling.

**Figure 5:**
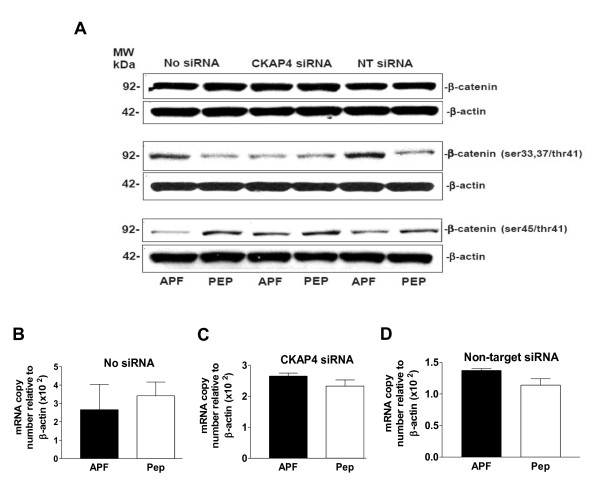
**β-catenin phosphorylation in T24 bladder cancer cells**. **A**, Western blot analysis of β-catenin protein expression and phosphorylation activity in cells electroporated in the presence of no siRNA (Lanes 1 and 2), CKAP4 siRNA (Lanes 3 and 4), or scrambled non-target (NT) siRNA (Lanes 5 and 6), and treated with *as*-APF (APF) or its inactive control peptide (Pep). β-actin served as a standard control. **B**, Quantitative real time RT-PCR analysis of β-catenin mRNA expression in T24 cells electroporated with no siRNA, **C**, CKAP4 siRNA, or **D**, non-target siRNA, and then treated with *as*-APF (APF) or its inactive control peptide (Pep). Each experiment was performed in duplicate on at least three separate occasions. Data are expressed as mean ± SEM.

We therefore proceeded to examine the effects of *as*-APF on β-catenin and β-catenin phosphorylation in T24 cells. As shown in Figure 5A, although subtle increases in β-catenin phosphorylation were apparent following APF treatment of nontransfected cells when antibodies against phosphoserine 33, 37 and threonine 41 (ser33,37/thr41) sites were used, there was no apparent change in total cell β-catenin protein. In addition, decreased phosphorylation was apparent following APF treatment when antibodies that recognized phosphoserine 45 (ser45) and phosphothreonine 41 (thr41) were used. Again, these changes in phosphorylation were abrogated by CKAP4 knockdown, and there were no significant differences in β-catenin mRNA levels regardless of transfection status (Figure 5B-D) (p > .05 for all PCR comparisons, including target gene mRNA relative to β-actin or GAPDH mRNA; data shown for normalization to β-actin expression, only). Although these findings suggest subtle changes in β-catenin phosphorylation in response to APF, they also provide additional evidence that APF may mediate its profound effects on cell proliferation and gene expression via means other than (or in addition to) regulation of canonical Wnt/frizzled signaling pathways.

### Downregulation of MMP2 expression by APF in T24 bladder cancer cells via CKAP4

Wnt/frizzled signaling is also known to stimulate cellular production of specific gelatinases including MMP2 [31,32] which has been implicated in HB-EGF activation and cleavage [33] as well as the progression and/or occurrence of various cancers including bladder cancer [34-37]. As the expression of MMP2 is also known to be stimulated by HB-EGF in carcinoma cells [38], we next determined whether *as*-APF also regulated MMP2 expression in T24 cells.

**Figure 6:**
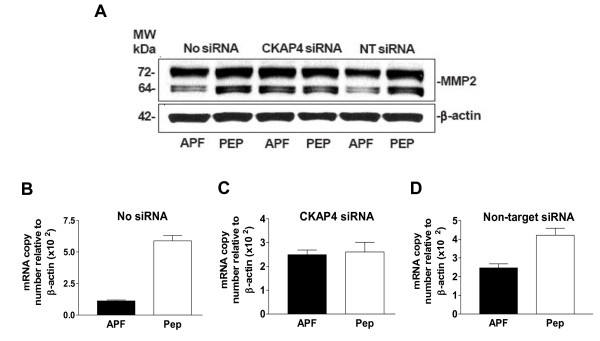
**MMP2 expression in T24 bladder cancer cells**. **A**, Western blot analysis of MMP2 protein expression in cells electroporated in the presence of no siRNA (Lanes 1 and 2), CKAP4 siRNA (Lanes 3 and 4), or scrambled non-target (NT) siRNA (Lanes 5 and 6), and treated with *as*-APF (APF) or its inactive control peptide (Pep). β-actin served as a standard control. **B**, Quantitative real time RT-PCR analysis of MMP2 mRNA expression in T24 cells electroporated with no siRNA, **C**, CKAP4 siRNA, or **D**, non-target siRNA, and then treated with *as*-APF (APF) or its inactive control peptide (Pep). Each experiment was performed in duplicate on at least three separate occasions. Data are expressed as mean ± SEM.

As shown in Figure 6A, APF treatment decreased MMP2 protein expression in nontransfected or non-target siRNA-transfected, but not CKAP4 siRNA-transfected, T24 cells. Similarly, APF treatment resulted in significantly decreased MMP2 mRNA levels in nontransfected or non-target transfected but not CKAP4 siRNA-transfected cells (Figure 6B-D) (p < .01 for nontransfected and p < .05 for non-target transfected cells, regardless of whether target gene mRNA expression was calculated relative to β-actin or GAPDH mRNA; data shown for normalization to β-actin expression, only). These findings indicate that APF inhibits MMP2 mRNA and protein expression in T24 cells via CKAP4.

## Discussion

The current study shows that APF mediates its antiproliferative effects in T24 bladder carcinoma cells via the CKAP4 transmembrane receptor, as found previously for normal bladder epithelial cells [27]. Further, it indicates that the mechanism whereby APF inhibits bladder carcinoma cell proliferation via CKAP4 involves the regulation of phosphorylation (with activation or inactivation) of various cell signaling molecules including Akt, GSK3β, β-catenin, along with mRNA and protein expression of p53 and MMP2.

CKAP4, which was first described as a reversibly palmitoylated type II transmembrane receptor [28], was previously shown to bind the synthetic form of a natural bladder epithelial cell antiproliferative factor (*as*-APF) and mediate its effects on normal bladder epithelial cell proliferation [27]. Results from the current study show that the CKAP4 receptor is also required for inhibition of bladder carcinoma cell proliferation by *as*-APF *in vitro*. In addition, experiments performed to elucidate the mechanism of APF activity indicate that this frizzled 8-related glycopeptide induces altered expression or phosphorylation of certain proteins that differ in some aspects from those seen in canonical Wnt/frizzled signaling.

Downstream signal transducers for Wnt/frizzled signaling include Akt, GSK3β, and β-catenin [39]. The serine threonine kinase Akt, also known as protein kinase B (PKB), is a central regulator of cell proliferation, motility and survival whose activity is often altered in human malignancies [40]. Akt mediates its downstream effects via phosphorylation/inactivation of GSK3β ser9, with subsequently decreased phosphorylation of the GSK3β target β-catenin, resulting in increased β-catenin nuclear translocation, binding to T-cell factor, and stimulation of gene expression related to cell proliferation and survival [30,41]. In addition to its association with malignant cell proliferation, increased Akt phosphorylation/activation has also been linked to the invasive properties of bladder cancer cells [40]. The inhibition of Akt ser473 and thr308 phosphorylation by APF suggests that APF may profoundly inhibit bladder epithelial cell Akt activity, and therefore decrease bladder carcinoma cell invasive potential, as well.

GSK3β activity is reduced by phosphorylation of ser9 but stimulated by phosphorylation on tyr216 [42], and the downstream effects of Akt activation/phosphorylation during Wnt/frizzled signaling include increased ser9 phosphorylation with decreased activity of GSK3β, decreased GSK3β-inducedβ-catenin ser33,37 phosphorylation, and subsequently decreased β-catenin ubiquitination and degradation. If *as*-APF mediated its activity in T24 cells purely by inhibiting canonical Wnt/frizzled signaling (like other secreted frizzled-related cell growth inhibitors), GSK3β ser9 phosphorylation should have been decreased substantially, while tyr216 phosphorylation (which may be mediated by mitogen-activated protein kinase kinase (MEK) 1/2) [43] should not have been affected. Our results, which showed only a very minimal decrease in GSK3β ser9 phosphorylation, but a substantial decrease in GSK3β yr216 phosphorylation, indicate that *as*-APF: 1) does not mediate its activity purely by regulating Wnt/frizzled canonical signaling; 2) may inhibit GSK3β and additional kinases (such as MEK 1/2); and 3) may mediate its antiproliferative effects in T24 cells via inhibition of Akt, GSK3β, and/or MEK1/2 involving downstream effects on targets in addition to β-catenin. Indeed, the modest increase in apparent phosphorylation of β-catenin ser33,37/thr41, along with a decrease in phosphorylation of β-catenin ser45 (which is mediated by autophosphorylation, casein kinase 1, and/or a complex of cyclin D1/cyclin-dependent kinase 6, and which primes ser33,37 for phosphorylation by GSK3β) [42,44], suggests that the regulation of total β-catenin protein and/or inhibition of canonical Wnt/frizzled signaling may not be the sole mechanism by which APF induces its effects on cell proliferation and gene expression.

Matrix metalloproteinases (MMPs) are a multigene family of zinc-dependent endopeptidases that degrade extracellular matrix components, whose expression is also regulated via Wnt/frizzled signaling pathways [31,32] and has been shown to correlate with invasive potential of many different tumors [45]. Expression of MMP2 is associated with bladder carcinoma cell invasion and metastasis [34-37]. The ability of *as*-APF to significantly inhibit MMP2 mRNA and protein expression in T24 cells also suggests that *as*-APF may be able to decrease the invasive potential of bladder carcinoma cells as well as inhibit their proliferation.

Previous experiments performed by Jayoung Kim showed that p53 mediated the antiproliferative effects of native APF in both normal and T24 bladder carcinoma cells [22]. The current study confirms this result by showing that synthetic *as*-APF also increases p53 protein and mRNA expression in T24 cells, and it further demonstrates the role of the CKAP4 receptor in APF-induced p53 upregulation.

Although the expression or activation of each of the cell proteins shown to be modified by APF can be regulated via Wnt/frizzled pathways, the specific alterations seen in Akt/GSK3β/β-catenin phosphorylation and the lack of an effect of APF on total cellular β-catenin levels suggest that this secreted frizzled-related peptide does not inhibit T24 bladder cell proliferation solely via inhibition of canonical Wnt/frizzled signaling. Whether the CKAP4 receptor can mediate transmembrane signaling, and/or whether it functions as a chaperone protein for cytoplasmic or nuclear translocation of APF, is unknown [27,29]. However, the myriad effects of APF on cell protein activation and expression discovered in the current as well as previous studies [19,21] indicate it may inhibit cell proliferation by regulating the activity of more than one signaling pathway or transcriptional regulatory factor.

The ability of *as*-APF to inhibit GSK3β tyr216 phosphorylation without inhibiting GSK3β ser9 phosphorylation suggests it may also be a potent GSK3β enzyme inhibitor in T24 cells. Recent studies on natural compound GSK3β inhibitors suggest that this class of drugs may be promising for the regulation of certain cancers [46]. Additional *in vitro *and *in vivo *studies with this intriguing natural frizzled 8-related glycopeptide are in progress to elucidate further its important cell regulatory function(s) as well as its potential as a therapeutic agent.

## Abbreviations

APF: antiproliferative factor; *as*-APF: asialo-APF; CKAP4: cytoskeleton-associated protein 4; IC: interstitial cystitis; FBS: fetal bovine serum; qRT-PCR: quantitative reverse transcriptase polymerase chain reaction; Rb: retinoblastoma gene; HB-EGF: heparin-binding epidermal growth factor-like growth factor; siRNA: small interfering ribonucleic acid; MMP: matrix metalloproteinase; GSK: glycogen synthase kinase; Wnt: wingless-int; Akt: AKR-transforming enzyme; NT: non-target; GAPDH: glyceraldehyde phosphate dehydrogenase; ser: serine; tyr: tyrosine; thr: threonine; siRNA: small interfering RNA

## Competing interests

SKK is named as an inventor on a patent for APF and a patent application that includes synthetic *as*-APF.

## Authors' contributions

HMS carried out major experiments for these studies. KRK and COZ performed some of the qRT-PCR, and LG and COZ performed some of the Western blots, for this paper. SKK supervised the research and interpretation of the data. HMS and SKK also prepared the manuscript, which was reviewed by the other authors prior to submission.
